# Returning of antiretroviral medication dispensed over a period of 8 months suggests non-adherence despite full adherence according to real time medication monitoring

**DOI:** 10.1186/s12981-020-00313-z

**Published:** 2020-09-10

**Authors:** Kennedy Michael Ngowi, Lydia Masika, Furaha Lyamuya, Eva Muro, Blandina T. Mmbaga, Mirjam A. G. Sprangers, Pythia T. Nieuwkerk, Rob E. Aarnoutse, Peter Reiss, I. Marion Sumari-de Boer

**Affiliations:** 1 Kilimanjaro Clinical Research Institute, Kilimanjaro Christian Medical Centre, Moshi, United Republic of Tanzania; 2 Department of Medical Psychology, Amsterdam University Medical Centers, Amsterdam, The Netherlands; 3 Kilimanjaro Christian Medical Center, Moshi, United Republic of Tanzania; 4 Kilimanjaro Christian Medical University College, Moshi, United Republic of Tanzania; 5 Radboud Institute for Health Sciences & Department of Pharmacy, Radboudumc, Nijmegen, The Netherlands; 6 Department of Global Health and Division of infectious Diseases, Amsterdam Institute for Global Health and Development, Amsterdam University Medical Centers , Amsterdam, The Netherlands; 7 Stichting HIV Monitoring, Hogeschool van Amsterdam, Amsterdam, The Netherlands; 8 Department of Internal Medicine: Infectious Diseases, Radboudumc, Nijmegen, The Netherlands

**Keywords:** Adherence, ART, mHealth, Realtime medication monitoring, Case report

## Abstract

Real-time medication monitoring (RTMM) may potentially enhance adherence to antiretroviral treatment (ART). We describe a participant in an ongoing trial who, shortly after completing trial participation, died of cryptococcal meningitis despite high levels of adherence according to self-report, pill-counts and RTMM (> 99%). However, she evidenced consistently high HIV viral load throughout the 48-week study follow-up. Subsequently, her relatives unsolicitedly returned eight months’ dispensed ART medication that she was supposed to have taken. This brief report illustrates the challenges of adherence measurements including RTMM, and reinforces the need to combine adherence assessments with viral load monitoring in HIV care.

## Introduction

Sustained adherence to antiretroviral treatment (ART) at levels over 95% among people living with HIV (PLHIV) is required in order to prevent treatment failure and the emergence of drug resistance [1]. Therefore, monitoring of adherence is of paramount importance in the management of PLHIV. In resource-limited settings, self-reported adherence and adherence calculations based on pharmacy refill counts are often used, but have been shown to overestimate actual adherence rates [2]. Several reasons for such over-reporting have been found, including: (1) fear of being expelled from the study and losing the benefits provided by study participation, (2) social distance between study nurses and participants causing mistrust, (3) nursing staff that is perceived as condemning being non-adherent, and (4) counselling provided by study staff that may discourage openness, or alternatively, may be too permissive making misreporting easy [3, 4]. Electronic monitoring devices consisting of either pill bottle caps or pillboxes that record and store the date and time of each opening of the cap or box are generally considered to be the most accurate and reliable adherence assessment method. The assumption underlying electronic medication monitoring is that each pill bottle or pill box opening represents ingestion of medication [5–7]. Here we present the case of a woman living with HIV that challenges this assumption. We first describe the ongoing randomized clinical trial in which the subject had participated.

## Trial

The trial aims to investigate the effect of two mobile health (mHealth) strategies, real time medication monitoring (RTMM) and use of short message service (SMS), on adherence to treatment among PLHIV in Kilimanjaro, Tanzania. A total of 249 adults on ART who were subjectively judged to be non-adherent by nurse counsellors were recruited from two health centres. The judgement was based on missed clinic visits, reported non-adherence and returning of leftover pills to the clinic. Participants were randomized equally in a ratio of 1:1:1 to receive SMS, RTMM or standard care and followed for 48 weeks. The study was approved by the Kilimanjaro Christian Medical College Research Ethics and Committee (CRERC) and the National Health Research Ethics Committee (NatHRec) of Tanzania.

Our case was randomized to the RTMM arm in which participants use an RTMM-device, the so-called Wisepill device, to take their medication. Each opening of the box is recorded, including the time stamp and status of the battery. This information is instantly sent through mobile data using General Packet Radio Service (GPRS) to a central database which has a secure web-based interface where the usual time of intake (agreed time between patient and healthcare provider) is stored. If the box is not opened on time, the participant receives a short message service (SMS) text on their mobile phone which acts as a reminder to take medication. The RTMM system generates a report that shows pill box openings. This report is then used during bi-monthly consultations with study nurses to discuss adherence and strategies for improvement, if needed. In the trial, adherence in all arms is measured through self-reporting by asking about the number of missed pills during the past month and through pharmacy refill counts by counting the number of leftover pills at each visit. In the RTMM arm, it is also measured through RTMM, in which pill-box openings are considered to indicate medication intakes.

## Case

A 38-year old single woman was first diagnosed with HIV in April 2017 and started on ART (tenofovir + lamivudine + efavirenz) in June 2017. Her medical file showed that she had a viral load of 423,000 copies/ml in April 2017 and a CD4-count of 276 cells/µL in June 2017. She was enrolled in our study in February 2018 and at that time was still on the first-line antiretroviral regimen of efavirenz, lamivudine and tenofovir. At enrolment, she had a high viral load of over 200,000 copies/ml, indicating virological failure possibly as a result of treatment non-adherence. As part of participating in the trial (RTMM arm), she reported detailed information about her adherence to treatment during clinic visits. She consistently reported to take all her antiretroviral pills except for one visit where she mentioned to have missed a few pills in the previous month. Her viral load continued to be high (> 200,000 copies/ml) for more than a year during follow-up in our trial despite extensive adherence counselling during study visits. From January 2019 onward, her health gradually deteriorated. She started feeling sick and cryptococcal meningitis was diagnosed. At her last 48 weeks study follow-up visit in February 2019, she looked fatigued and weak. As part of the trial, an exit-interview was scheduled to obtain information about her experience on the usage of RTMM. She did not attend this interview. The patient died of cryptococcal meningitis in July 2019, in spite of having been switched to a second-line antiretroviral regimen of atazanavir, abacavir and lamivudine.

## Virological and immunological outcomes

The participant had a viral load of 231,037 copies/ml at enrolment in February 2018. Three months later in May 2018, her CD4 cell count was 56 cells/μL. When the viral load measurement was repeated in August 2018, it remained high at 262,000 copies/mL and again in January 2019 (232,352 copies/mL) and February 2019 (245,932 copies/mL). At the last study visit in February 2019, her CD4-count had decreased to 16 cells/μL. In May 2019, her viral load had decreased to 44 copies/mL and her CD4 count increased to 160 cells/μL.

## Adherence to treatment

**Fig. 1:**
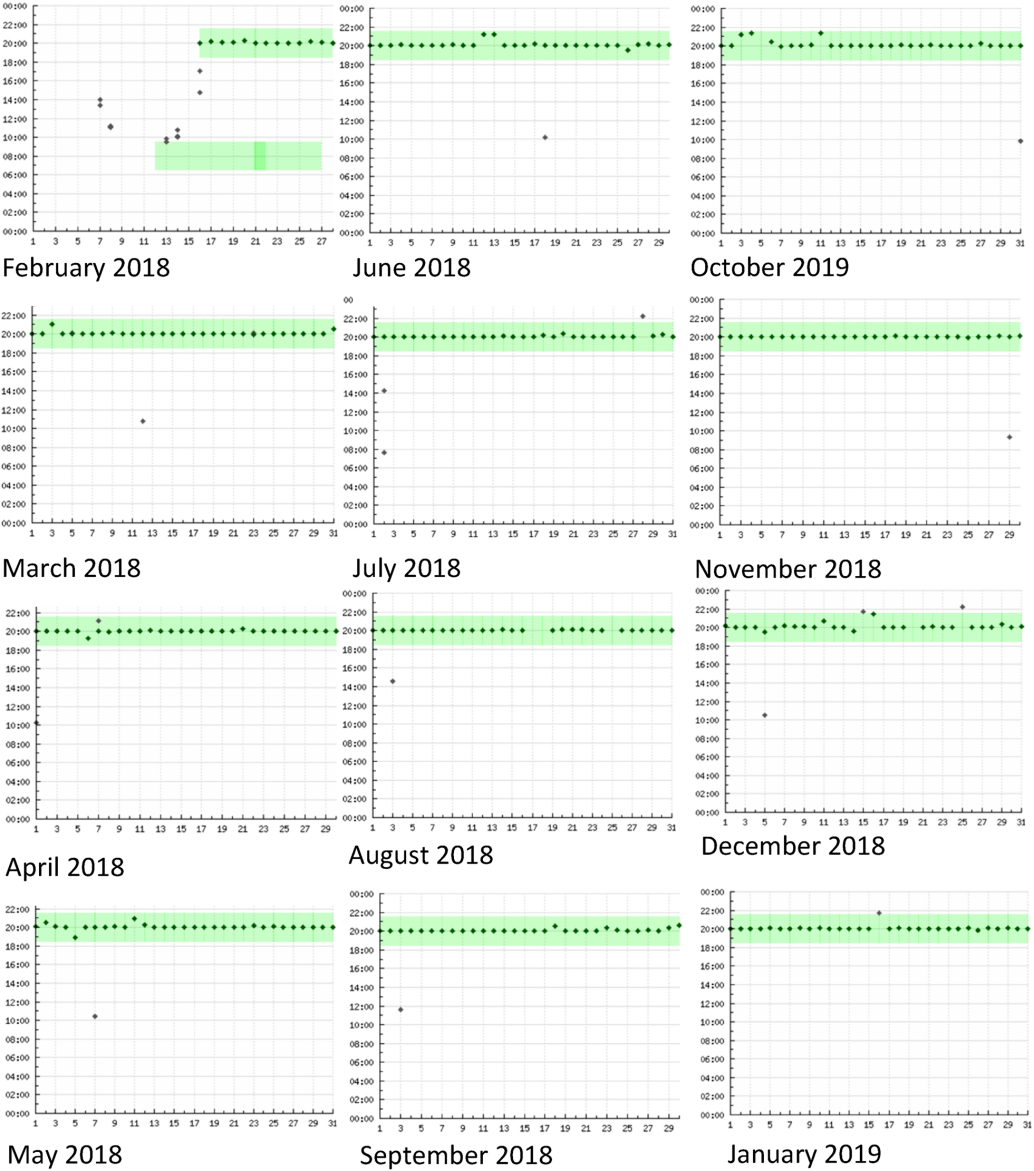
Adherence graphs (X-axis is the day of the month, Y-axis is the time of intake whereby the dots display the opening of the pillbox and the bars show the agreed time of intake)

**Fig. 2:**
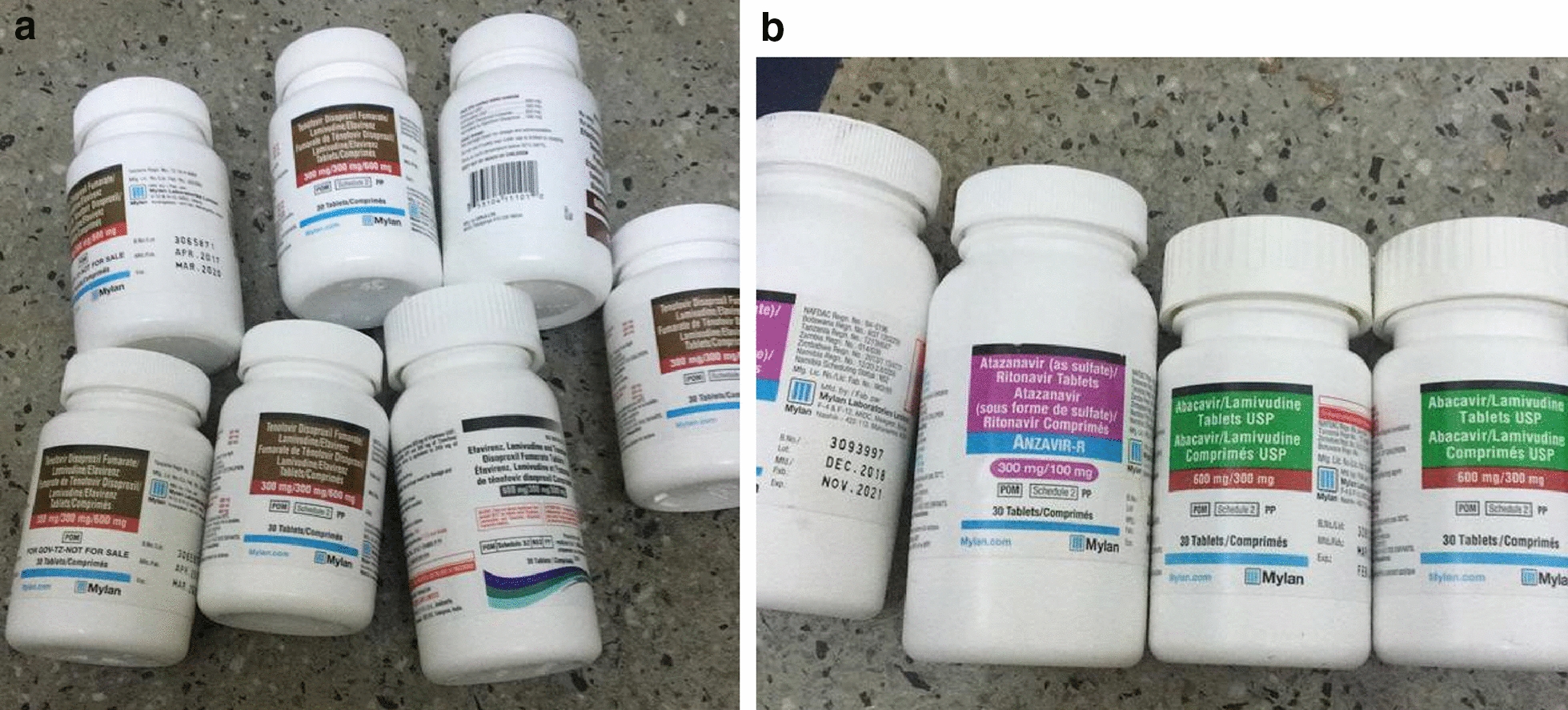
**a** First-line Medication that was returned by relatives of our trial participant. **b** Second-line Medication that was returned by relatives of our trial participant

The participant’s adherence rates according to self-reporting, pharmacy refill monitoring, and RTMM were recorded at clinic visits during the study period. She reported to only have missed 2 pills in the total follow up of 355 days, which corresponds to an adherence rate of 99%. This was confirmed by pharmacy refill counts, according to which adherence over the total period was likewise above 99%. The high adherence rate of 99% was further confirmed by RTMM. Figure 1 shows the monthly adherence graphs that were generated by the RTMM device. The graphs show that most of the time, the patient consistently opened the RTMM device around 8 pm. In the feedback sessions where she received tailored feedback on her adherence reports, she always confirmed that she took all her medication adequately. Only at one visit in July 2018, she mentioned that her intake was not adequate. In addition, she explained that she used her phone alarm as an extra reminder and that her mother also helped reminding her to take medication. She confirmed that she experienced no problems in taking medication. After finishing her last study visit (end of study follow-up), she was invited for an exit-interview which she was not able to attend (Fig. 2).

## Leftover pills

After our participant had died, her relatives came unsolicited to bring back leftover medication. In total, pills dispensed for 151 days (45% of the study period) for the first-line regimen were returned, and pills dispensed for 60 days for the second-line regimen were returned (43% of second-line regimen duration). The production dates of the returned first-line pills ranged from March 2017 to March 2018, indicating that those were the pills she had been supposed to be taking during the study. We had to conclude that despite the excellent adherence rates according to all three measures, i.e. self-report, pharmacy refill monitoring, and RTMM, her adherence had actually been low.

This case report describes a woman living with HIV whose adherence indicators showed excellent adherence but were in sharp contrast with her actual health indices. This case report illustrates that opening of the pillbox, the so-called medication event, does not necessarily mean actual pill intake. We can only speculate what made her open the pillboxes but not ingest the medication. Perhaps she wanted to please the researchers and study nurses, or prevent receiving an SMS reminder post-intake-time, or possibly avoid adverse reactions from those close to her. Despite extensive counselling and discussions on her clinical outcomes and adherence as part of the study, the nurse counsellors who provided feedback on the adherence reports were not aware of signals or information of her non-adherence. In addition, she had disclosed her status to relatives, and reported not to have experienced stigmatization but rather received a lot of social support.

Previous studies have shown that opening the device is a good proxy of real intake of pills at the group level [3, 7], but our case clearly illustrates that exceptions exist at the individual level.

The question arises under what circumstances real-time monitoring is most useful. A study in Uganda found that real-time monitoring was only effective in the early stages of ART treatment, or if patients show medication side-effects [8]. Another study from Botswana also showed that RTMM is more effective in improving adherence for patients novel to ART, compared to experienced and knowledgeable patients [9]. A third study showed that using real-time monitoring alone had a less positive impact than when an SMS reminder was sent before the actual time of intake [10]. Such an SMS is particularly helpful if it contains educational and/or motivational information to stimulate medication intake (Table 1).

**Table 1:** Visit dates, adherence measures, virological, immunological and clinical findings

Visits	a	Self-report (%)^a^	Pharmacy Refill (%)	RTMM (%)	Viral load (copies/mL)	CD4 count (cells/μL)	Signs and symptoms
Screening	15-1-2018						NR
Enrolment	16-2-2018	100	90	N/A	231,037	NM	NR
Extra visit	12-3-2018	100	100	100		NM	NR
Visit 2	7-5-2018		161	100	NM	56	No any complaints
Visit 3	2-7-2018	100	100	100	NM	NM	Healthy looking
Visit 4	9-8-2018	100	100	100	NM	NM	Good condition
Extra visit	29-8-2018	93	90	85	262,000	NM	NR
Visit 5	3-10-2018		137	100	NM	NM	Active and healthy
Extra visit	31-10-2018	100	146	96			NR
Visit 6	5-12-2018		111	100	NM	NM	Healthy looking
Extra visit	9-1-2019	100	100	100	232,352	NM	NR
Visit 7	6-2-2019		96	97	245,932	16	Looks sick (weak and fatigue)
Extra visit	29-5-2019				44	160	

^a^Based on missed pills in the past month

Various studies have shown benefits of adherence-enhancing interventions based on electronic medication monitoring (6–8). However, this case helps to remind us that despite high levels of electronically monitored and self-reported adherence, actual adherence may be poor for some individuals. The VOICE-D trial, which is a post-trial study asking participants why their actual product use was lower than they had reported, is worth mentioning. The results suggest that participants, acknowledged the importance of daily monitoring and honest reporting, and yet still over-reported their adherence due to fear of being expelled from the study and worry to be misjudged by nurses for criticizing the interventions [3]. Apparently, our participant refused to take ART but was unwilling or felt uncomfortable to communicate this to her health care providers. It serves as a reminder of the importance of making patients feel free to discuss their concerns and wishes with their healthcare providers, without the fear of being judged or blamed for being non-adherent or refusing medication.

In conclusion, this report illustrates that a valid and accurate measurement of medication adherence remains challenging, and reinforces the importance of combining the assessment of adherence with viral load monitoring as part of HIV care.

## Data Availability

All the Data are available and will be shared after signing of the Data Transfer agreement between Sender and Receiver .
